# Electrochemical Measurement of Noscapine and Lorazepam Using a Carbon Paste Electrode Modified with Multi-walled Carbon Nanotubes and Natural Deep Eutectic Solvent

**DOI:** 10.22037/ijpr.2021.114557.14911

**Published:** 2021

**Authors:** Maryam Vafadar, Ebrahim Zarei, Alireza Asghari

**Affiliations:** a *Department of Chemistry, Semnan University, Semnan, Iran. *; b *Department of Basic Sciences, Farhangian University, Tehran, Iran.*

**Keywords:** Simultaneous determination, Noscapine, Lorazepam, Modified electrode, Pharmaceutical samples

## Abstract

In the present study, simultaneous voltammetric determination of noscapine (NOS) and lorazepam (LOR) was studied for the first time. A carbon paste electrode modified with multi-walled carbon nanotubes (MWCNTs) and natural deep eutectic solvent (NANADES) (MWCNTs/NADES/CPE) was used for this purpose. Electrochemical impedance spectroscopy (EIS) was applied for the investigation of the electron transfer rate of [Fe(CN)6]^3-/4-^ as a redox couple probe on the surface of the MWCNTs/NADES/CPE. The modified electrode preserved and combined the properties of the individual modiﬁers synergistically. A signiﬁcant enhancement in the peak current responses of NOS and LOR was observed on the modiﬁed electrode surface compared to the bare electrode. Under the optimal conditions, the peak current of differential pulse voltammograms was linearly dependent on analyte concentration in the range of 3-1700 µM for NOS and 1-2220 µM for LOR. The limit of detection (LOD) for NOS and LOR was 1.90 µM and 0.69 µM, respectively. Finally, this strategy was also employed for the determination of NOS and LOR in pharmaceutical samples.

## Introduction

Abbott et al. proposed deep eutectic solvents (DESs) in 2002, for the first time (1). DESs are potential alternative solvents for ionic liquids that have similar properties to ionic liquids but are less toxic, cheaper to produce, and often biodegradable (2). Natural deep eutectic solvents (NADESs) as a new type of DESs were presented when Choi et al. in 2011 resulted in many plant primary metabolites forming a DES-like liquid when mixed in certain conditions combinations (3). NADESs can be deﬁned as a mixture of two or more natural organic compounds when at a particular molar ratio, having a melting point signiﬁcantly lower than that of either individual component (4). The compounds interact together via intermolecular hydrogen bonds, in the absence of chemical reactions, one acting as the hydrogen bond donor and the other as the acceptor (5). The fabrication process is usually achieved after a physical mixture of the components, which heating and vigorous physical mixing can accelerate this process (5). The most encountered NADESs is based on a mixture of choline chloride as a hydrogen bond acceptor and two or more hydrogen bond donors such as some amino acids, organic acids, polyols, and sugars (6). 

Also, there is a remarkable interest in using carbon nanotubes (CNTs) as an electrode modifier in sensors due to their attractive properties, such as high chemical stability, very high mechanical strength, and excellent electrical conductivity (7). The subtle electronic behavior of CNTs as molecular-scale wires indicates that they can increase electron-transfer reaction when applied as electrode materials (8-10). 

**Scheme 1:**
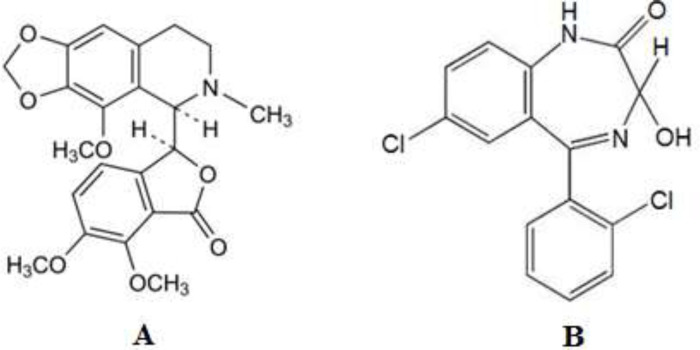
Structures of (A) NOS and (B) LOR

Noscapine (NOS) (Scheme 1A) with 2-8% concentrations is the second most abundant alkaloid in opium. It is commonly applied as an antitussive drug (11, 12). NOS has no analgesic activity unlike morphine and codeine. Its major pharmaceutical application is antitussive activity, which is equivalent to codeine reported by researchers (11). Recent investigations show that NOS can be a factor in causing apoptosis in many cells. The effective antitumor property of NOS on bladder tumors in nude mice and solid murine lymphoid tumors (13) and cancer cell lines related to the human prostate (14) has also been demonstrated. Several strategies have been reported for the detection of NOS using high-performance liquid chromatography (HPLC) (15), liquid chromatography-tandem mass spectroscopy (16), chemiluminescence (17), and spectrophotometric (18). 

Lorazepam (LOR) (Scheme 1B) is a short-acting benzodiazepine that causes central nervous system depression. It is used to treat anxiety, insomnia with anxiety, and as an anticonvulsant (19). Several methods have been stated for the analysis of LOR, such as liquid chromatographic in body fluids (20-22), spectrophotometric (23), gas chromatography-tandem mass spectrometry (24), adsorptive stripping voltammetry (25), and HPLC-MS (26). It should be mentioned that the reported methods for the determination of NOS and LOR, such as HPLC and spectrophotometric techniques, require expensive instrumentation, which may not be available in many laboratories (such as mass spectroscopy). Also, the analysis time is long, and in some cases, special pretreatment is required before analysis. On the other hand, the voltammetric methods compared with reported chromatographic methods have lower matrix interferences, so they do not need a time-consuming extraction step (27). Thus, some methods have been developed for the determination of NOS and LOR using voltammetry (28-34). 

According to our knowledge, there is no report about the simultaneous voltammetric measurement of NOS and LOR in the literature. Herein, a new electrochemical sensor based on MWCNTs/NADES modified carbon paste electrode was prepared to improve these compounds’ determination. Thus, in this work, MWCNTs/NADES/CPE was used as an electrochemical sensor for NOS and LOR. The MWCNTs/NADES/CPE could remarkably enhance the electrochemical responses of ACP and TSA and, as a result, improve the sensitivity and selectivity of NOS and LOR detection. Moreover, the electrochemical sensor was successfully employed to determine NOS and LOR in commercial drugs.

## Experimental

*Materials and reagents*

All the reagents used in this work including, choline chloride, glucose, FeCl_3_, vitamins B_1_ and B_2_, ascorbic acid, HNO_3_ (67%), NaOH, H_3_PO_4_, H_2_SO_4_, KCl, and NaCl, were supplied from Merck Company. The MWCNTs with 10-15 nm in diameter and purity of more than 90% were obtained from Merck. All solutions were fabricated using double-distilled water. NOS and LOR were of analytical grade and were provided by Merck chemical company (Darmstadt, Germany). Orthophosphoric acid and its salts in the pH range 4.0-8.0 were used to prepare buffer solutions. Nujol and graphite powder (particle diameter 0.1 mm) from Fluka were used. 

*Apparatus*

Electrochemical data were obtained with a three-electrode system using a Potentiostat/Galvanostat (OrgaFlex 500, Franc). The three-electrode system was applied with an Ag/AgCl (saturated KCl) electrode, a Pt wire, and a CPE modified with MWCNTs and NADES as reference electrode, a counter electrode, and the working electrode, respectively. The reference and counter electrodes were from Metrohm. Fourier transform infrared (FT-IR) spectra were measured with KBr pellets in the range of 400-4000 cm^-1^ using a Shimadzu 8400s spectrometer. Electrochemical impedance spectroscopy (EIS) measurements were performed using an ac potential with 5 mV amplitude, including a 0.01-100000 Hz frequency range under open circuit potential conditions. A pH meter, PHS-3BWModel (Bell, Italy), with a glass combined electrode, was used for pH measurements. An ultrasonic bath (SW3, Switzerland) was used at a frequency of 50/60 kHz.

*Synthesis of NADES*

NADES was prepared following the method developed by Dai *et al.* (Dai *et al.* 2013). 0.25 g choline chloride, 0.3252 g glucose, and 0.057 g water were placed in a bottle containing a stirring bar and cap. Then, the mixture was heated for about 60 min in a water bath below 50 °C with agitation till a clear liquid was produced. 

*Fabrication of working electrode*

The MWCNTs/NADES/CPE was prepared by mixing certain amounts of MWCNTs, NADES, and graphite powder with Nujol oil as the pasting liquid and thorough hand mixing in a mortar and pestle for 20 min until a uniformly wetted paste resulted. Then, a portion of this modified carbon paste was packed into the end of a glass tube with an inner diameter of about 3 mm. A copper wire was inserted into the carbon paste to make electrical contact. If necessary, to obtain a new surface, an excess of the paste was pushed out of the tube and polished with a weighing paper. The MWCNTs/CPE, NADES/CPE, and unmodified CPE were also constructed in a similar way to be applied for the comparison. 

## Results and Discussion

*SEM characterization*

**Figure 1:**
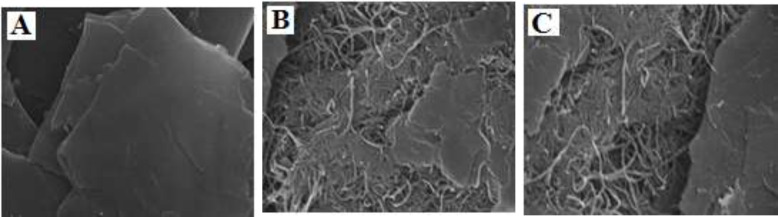
SEM image of (A) CPE, (B) MWCNTPE and (C) MWCNTs/NADES/CPE

Figure 1 shows SEM images for bare CPE, MWCNTs/CPE, MWCNTs/NADES/CPE. Results indicate that at a surface of CPE (Figure 1A), the layer of irregular flakes of graphite powder were present and isolated with each other. Figure 1B shows that after adding MWCNTs to the carbon paste matrix, MWCNTs with a specific three-dimensional structure exist on the surface of electrode MWCNTs/CPE. The presence of NADES had no significant effect on the morphology of the electrode surface (Figure 1C). This may be due to the small size of molecules choline chloride and glucose compared to MWCNTs.

*FT-IR spectra*

**Figure 2:**
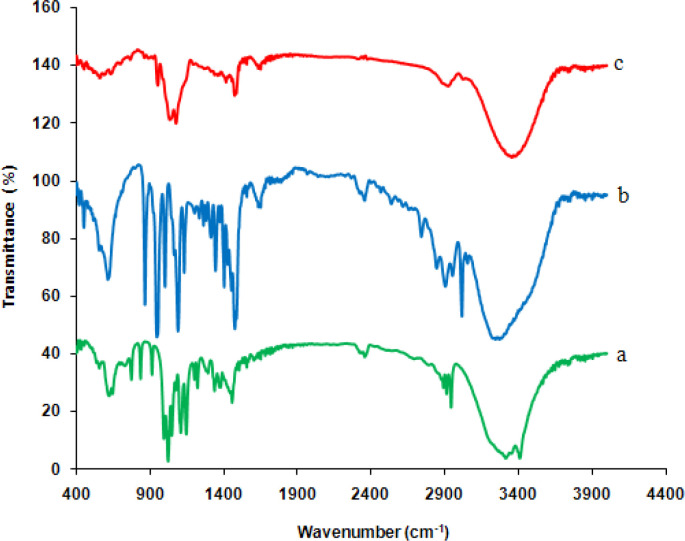
FTIR spectra of (a) glucose, (b) choline chloride, and (c) NADES

Figure 2 presents the FT-IR spectra of (a) glucose, (b) choline chloride, and (c) synthesized deep eutectic solvent. The spectra of glucose indicate a band at 3350 cm^-1^, which is related to O–H stretching vibration. The bands in the region the 1400-1199 cm^-1^ are attributed to O–C–H, C–C–H, and C–O–H bending vibrational modes, while those in the 900-1153 cm^-1^ region are due to C–O, and C–C stretching modes of the carbohydrates (35). In the choline chloride spectra, the OH vibrational stretching is seen at 3411 cm^-1^. Since choline chloride possesses both hydrogen bonding acceptors and donors, consequently, the presence of (most probably) intramolecular hydrogen bond affected the shape of the band; the existence of hydrogen bonds is also confirmed by the weak peak at 2889 cm^-1^ (36). In addition, the band at 3199 cm^-1^ is related to the *ν*(NH_3_^+^) vibrations of the charged amine derivatives, and the appropriate deformation vibration *δ*(NH_3_^+^) is positioned at 1616 cm^-1^ (37). In the FTIR analysis, the decrease in the amplitude of the wavenumber bands attributed to hydroxyl groups and also a slight shift in the O–H stretching vibration peak towards a lower value in the spectrum of NADES showed the formation of stronger H-bonds between glucose and choline chloride. Furthermore, the observed changes in the position, shape, and amplitude of other glucose and choline chloride bands can be related to this interaction.

*Impedance measurements*

**Figure 3:**
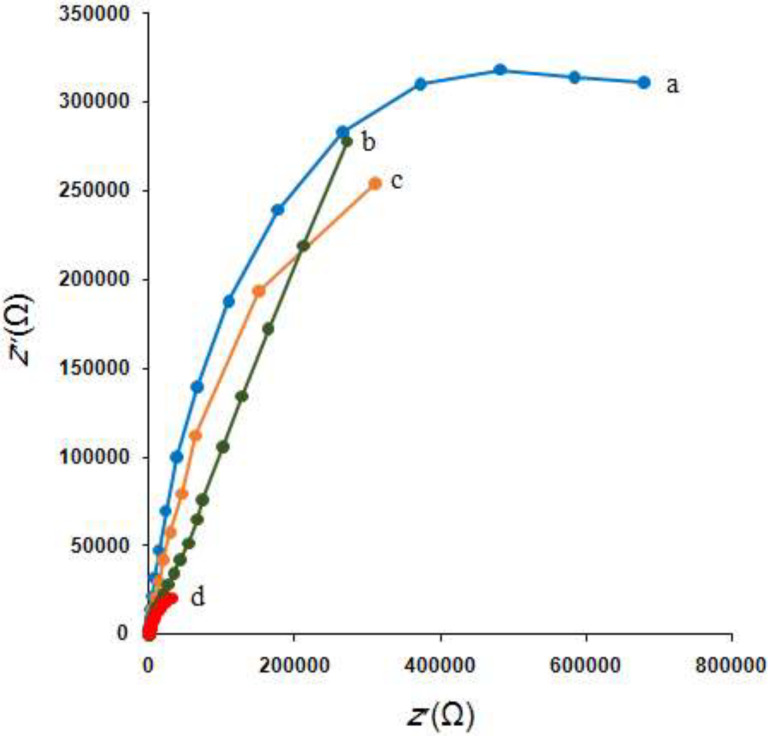
Nyquist plots of the (a) bare CPE, (b) MWCNTs/CPE, (c) NADES/CPE and (d) MWCNTs/NADES/CPE in 0.1 M KCl solution containing 1.0 mM [Fe(CN)_6_]^3-/4^. Conditions: *E*_ac_, 5 mV, frequency range, 0.1 to 10000 Hz

The MWCNTs/NADES/CPE was studied using electrochemical impedance spectroscopy (EIS). Figure 3 shows the typical results of AC impedance spectra of the bare CPE (curve a), NADES/CPE (curve b), MWCNTs/CPE (curve c), and MWCNTs/NADES/CPE (curve d) in 0.1 M KCl solution containing 1.0 mM [Fe(CN)_6_]^3-/4-^. In the full frequency range, EIS consists of a linear part and a semicircular part. The semicircle part results from the semicircular diameter of EIS and at higher frequencies is related to the electron-transfer resistance (*R*_ct_) and the electron-transfer limited process. The semicircle part of the curve has two intercepts. At the low-frequency intercept, the real axis value is the sum of the electron-transfer resistance (*R*_ct_) and the solution resistance (*R*_s_). At lower frequencies, the linear part is related to the diffusion process. It is obvious that significant differences were observed in the electrochemical impedance spectroscopy for these four electrodes. The bare CPE presented the largest semicircle in comparison to the other three electrodes with a large resistance of electron transfer in the high frequencies range, which indicates that [Fe(CN)_6_]^3-/4-^ has a low electrochemical activity at the unmodified CPE surface. However, compared with the bare CPE, NADES/CPE, MWCNTs/CPE, and MWCNTs/NADES/CPE showed that there is a semicircular section with a much smaller diameter in the high-frequency range, and this section can be related to proper ionic conductivity and consequently the lower resistance to electron transfer of the MWCNTs/NADES/CPE. These results show that the MWCNTs and NADES effectively modified the surface of CPE, and the conductivity was significantly increased.

*Electrochemical NOS and LOR sensor*

**Figure 4:**
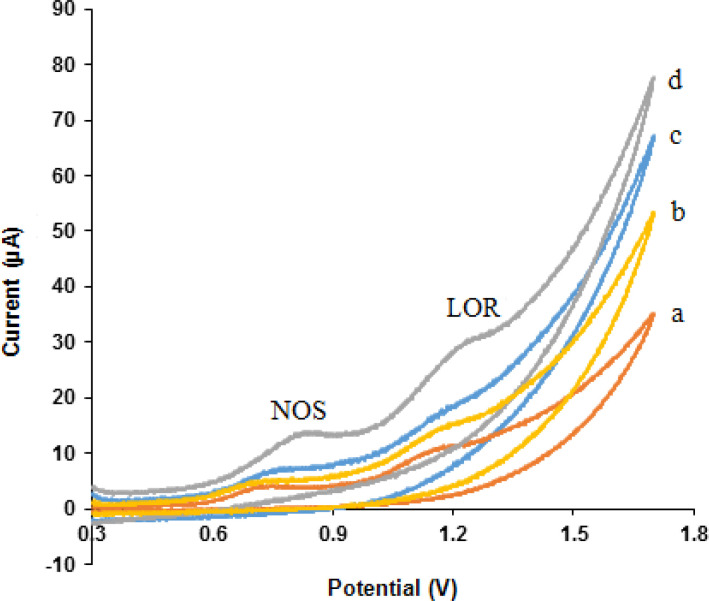
Cyclic voltammograms of NOS (500 µM) and LOR (500 µM) at the (a) bare CPE, (b) MWCNTs/CPE, (c) NADES/CPE and (d) MWCNTs/NADES/CPE in phosphate buffer solution (pH 7.0) at scan rate 50 mV s^-1^

Cyclic voltammograms obtained at different electrodes in phosphate buffer solution (pH 7.0) containing 500 µM NOS and 500 µM LOR within the potential window of 300-1700 mV and at a scan rate of 50 mV s^-1^ are shown in Figure 4 NOS and LOR indicated well-behaved oxidation peaks at the MWCNTs/NADES/CPE (curve e) with oxidation peak potentials of 0.84 and 1.25 V, respectively. 

These results presented that the MWCNTs and NADES increased peak currents of NOS and LOR at the MWCNTs/NADES/CPE (curve d) compared the bare CPE (curve a), MWCNTs/CPE (curve b), and NADES/CPE (curve c) which was related to excellent characteristics of nanomaterials such as, high surface area, suitable electrical conductivity and more electroactive interaction sites which can result in extensive mass transport and easy access to active sites. Therefore, the simultaneous detection of NOS and LOR is feasible on the MWCNTs/NADES/CPE surface.

*Effect of* *MWCNTs* *percentage in the* *MWCNTs/NADES/CPE*

**Figure 5:**
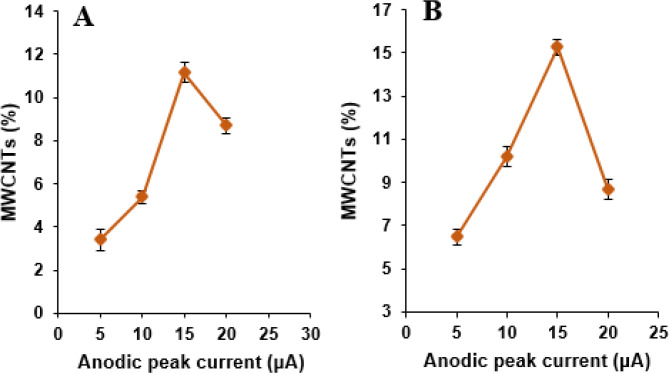
The dependence of the anodic peak currents of cyclic voltammograms of (A) NOS (500 µM) and (B) LOR (500 µM) on MWCNTs content at the MWCNTs/NADES/CPE surface (scan rate 50 mV s^-1^)

The effect of MWCNTs content as a modiﬁer in the carbon paste containing NADES content 10%, was ﬁrst investigated by varying its percentage as 5, 10, 15, and 20% with respect to graphite in phosphate buffer solution (pH 7.0) containing 500 µM NOS and 500 µM LOR (Figure 5). It was then seen that the oxidation peak currents for NOS and LOR increased with increasing in the MWCNTs percentage up to 15%; beyond this value, the anodic peak currents content of NOS and LOR decreases. It may be due to electrode conductivity decrease by the excessive amount of MWCNTs. Consequently, 15% MWCNTs were chosen as the optimum amount for the fabrication of the MWCNTs/NADES/CPE.

*Effect of* *NADES* *percentage in the* *MWCNTs/NADES/CPE*

**Figure 6:**
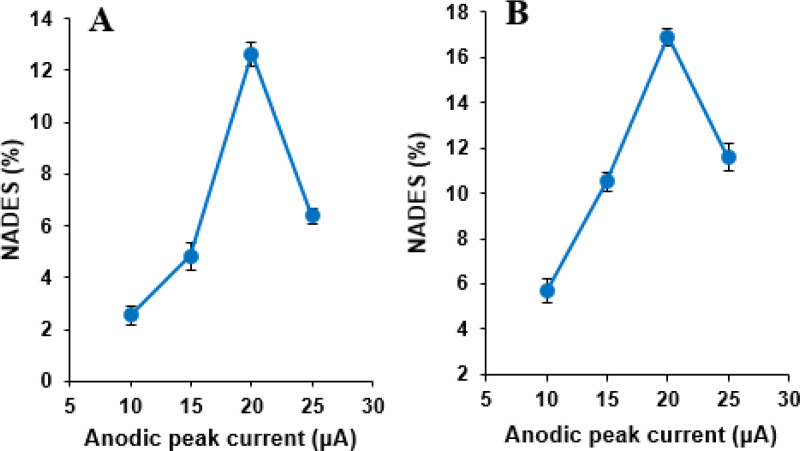
The effect of NADES content on the anodic peak currents of cyclic voltammograms of (A) NOS (500 µM) and (B) LOR (500 µM) at the MWCNTs/NADES/CPE surface (scan rate 50 mV s^-1^)

Voltammetric experiments were carried out with four different modified electrodes at MWCNTs content 15%, containing NADES ratio of 10, 15, 20, and 25% (w/w) with respect to the carbon paste in phosphate buffer solution (pH 7.0) containing 500 µM NOS and 500 µM LOR. The oxidation peak current values of cyclic voltammograms of NOS and LOR increased with the increase of NADES percentage, and the maximum value was obtained at 20% then decreased (Figure 6). It may be the fact that the presence of NADES increases the number of sites for NOS and LOR adsorption; subsequently, the oxidation peak current increases. However, the excess of NADES increases the resistance of the electrode, and the anodic peak currents of these compounds on the MWCNTs/NADES/CPE surface decreases. Therefore, 20% of NADES was selected as the optimum amount of NADES and was used to modify the CPE in this study.

*Influence of pH value*

**Figure 7:**
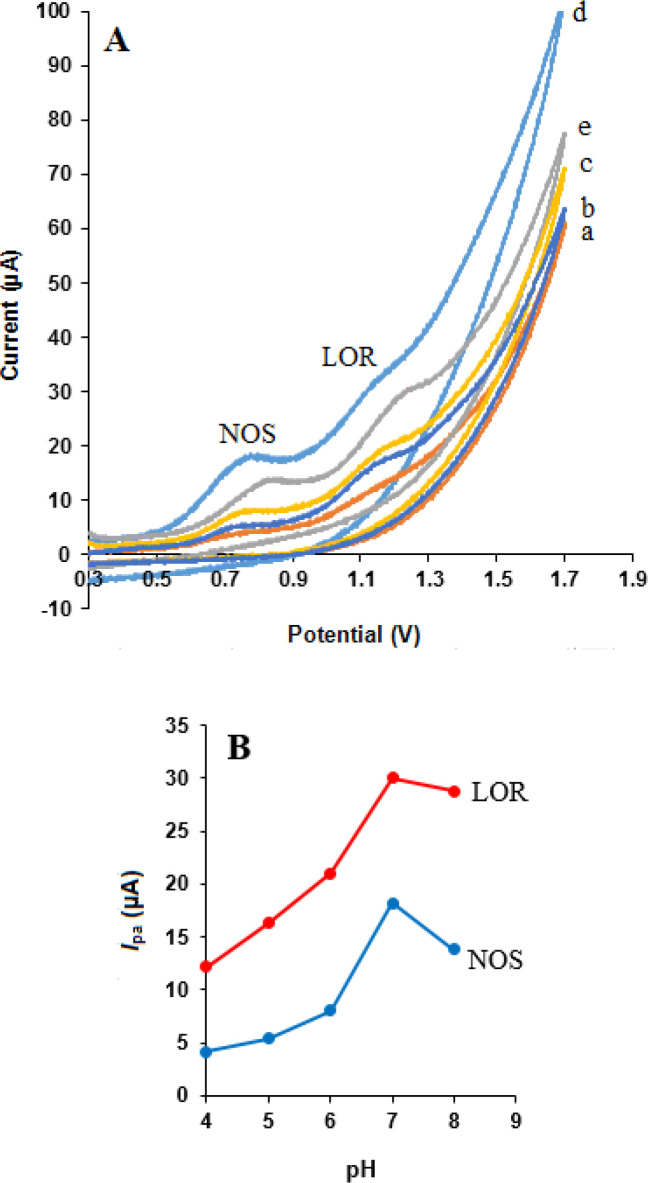
(A) Cyclic voltammograms of NOS (500 μM) and LOR (500 μM) in various pH values of buffer solutions: (a) 4.0, (b) 5.0, (c) 6.0, (d) 7.0 and (e) 8.0. (B) *I*_pa_-pH curves for oxidation of the drugs at the MWCNTs/NADES/CPE (scan rate 50 mV s^-1^).

Since the electrochemical behaviors of NOS and LOR are dependent on pH (29, 38), the effect of protons (H^+^) was investigated to optimize responses NOS and LOR at the surface of MWCNTs/NADES/CPE. Figure 7A shows the effect of buffer pH (from 4.0 to 8.0) on the response of 500 µM NOS and 500 µM LOR in phosphate buffer was studied at the surface of MWCNTs/NADES/CPE. According to Figure 7B, the oxidation peak currents of NOS and LOR were increased from pH 4.0 to 7.0 and then decreased at higher pH 7.0. Therefore pH 7.0 was selected as an optimum pH for other experiments. 

*Effect of scan rate*

**Figure 8:**
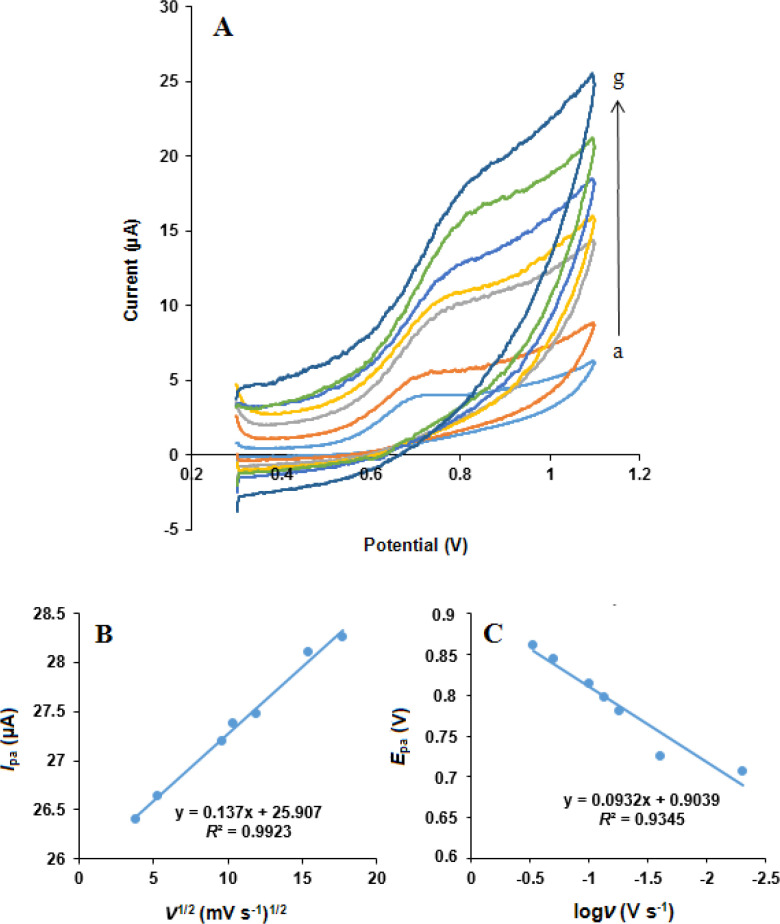
(A) Cyclic voltammograms of NOS (500 µM) at the MWCNTs/NADES/CPE surface in the buffer solution (pH 7.0) at different scan rates (a) to (g): 5, 25, 55, 75, 100, 200 and 300 mV s^-1^. (B) Dependence of anodic peak current on the square root of scan rate. (C) The linear relationship between logarithm of anodic peak potential and logarithm of scan rate

**Figure 9:**
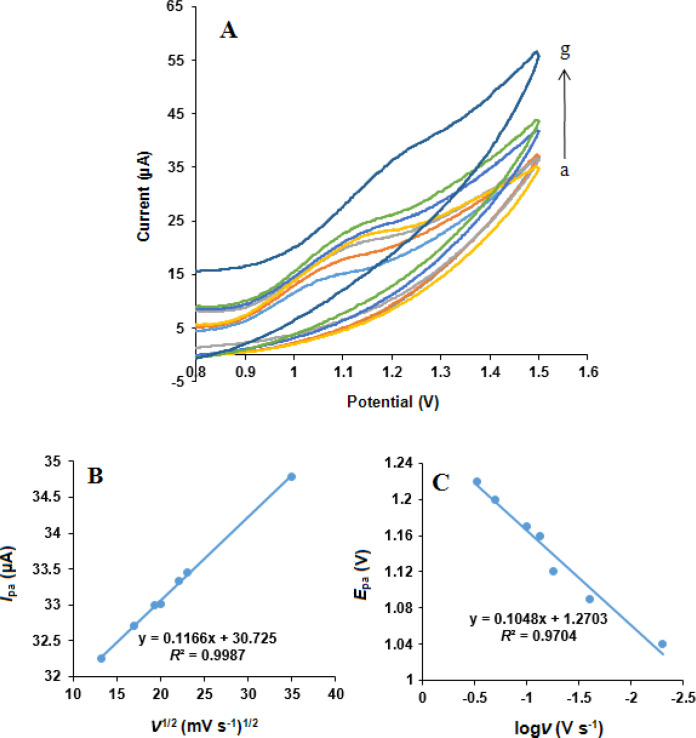
(A) Cyclic voltammograms of LOR (500 µM) at the MWCNTs/NADES/CPE surface in the buffer solution (pH 7.0) at different scan rates (a) to (g): 5, 25, 55, 75, 100, 200 and 300 mV s^-1^. (B) Dependence of anodic peak current on the square root of scan rate. (C) The linear relationship between the logarithm of anodic peak potential and logarithm of scan rate

The influence of scan rate on the simultaneous oxidation of NOS and LOR at the MWCNTs/NADES/CPE surface was studied using cyclic voltammetry in phosphate buffer (pH 7.0) at different potential scan rates, Figures 8 and 9. It can be observed that the oxidation peak currents for both NOS and LOR linearly increased with the square root of the scan rates (*ν*^1/2^), Figures 8B and 9B, suggesting which the redox reaction at the electrode surface is the predominantly diffusion-controlled process for both NOS and LOR (39). 

Moreover, the oxidation peak potential (*E*_pa_) of both NOS and LOR shifted in the positive direction with increasing scan rate. Also, the Tafel slope (Figures 8C and 9C) can be obtained from the slope of *E*_pa_ *vs*. log *ν* using the following equation (40):

E_p_ = b/2 log*ν* + constant 

Equation 1.

According to Equation 1, the slope of *E*_p _*vs*. logν curve is b/2, where b indicates the Tafel slope and b = 2 ×*E*_pa_/(logν). The Tafel slopes were found to be 0.186 and 0.209 V for NOS and LOR, respectively. According to Laviron, b = 2.303*RT*/*n*(1–*α*)*F*, for anodic peak, where *F* is the Faraday’s constant (96,485 C mol^-1^) (39) and the other symbols in the above equation have their usual meanings. In addition, based on the papers (29, 38), the number of transferred electrons (*n*) for both NOS oxidation and LOR oxidation is 2. Thus, the quantity of the electron-transfer coefficients of NOS and LOR (*α*) are 0.68 and 0.72, respectively.

The electrode surface coverage (Γ*) was measured from the linear part of the plot and by the following equation, which corresponds to a reversible process related to adsorbed species (40). 

I_p_ = (n^2^F^2^/4RT) νAΓ* 

 Equation 2.

where *I*_p_, *n*, *A*, and Γ* indicate the peak current, the number of electrons participating in the reaction (*n* = 1), the surface area of the electrode (0.28 cm^2^), and the surface coverage of the redox species, respectively.

*Chronoamperometric studies*

**Figure 10:**
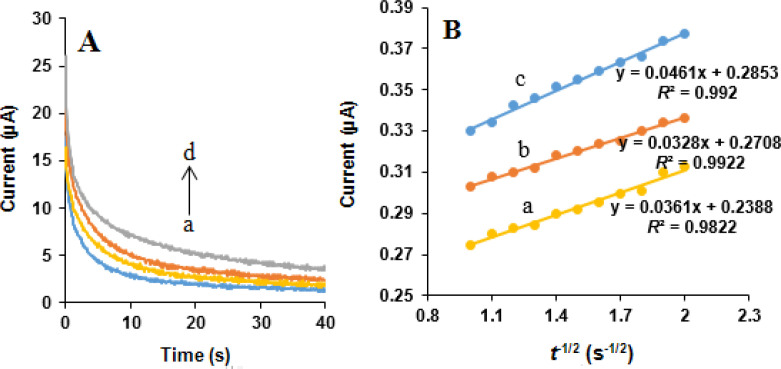
(A) Chronoamperograms obtained at the MWCNTs/NADES/CPE surface in the presence of (a) 0, (b) 400, (c) 500 and (d) 600 µM NOS in the buffer solution (pH 7.0) at setting of the working electrode potential at 850 mV *vs*. Ag/AgCl/KCl (3.0 M). (B) Cottrell’s plots for (a) 400, (b) 500 and (c) 600 µM NOS, based on the data obtained from the chronoamperograms

**Figure 11:**
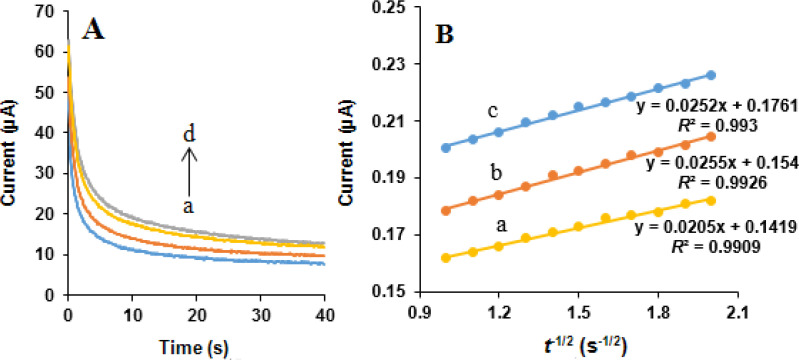
(A) Chronoamperograms obtained at the MWCNTs/NADES/CPE surface in the presence of (a) 0, (b) 400, (c) 500 and (d) 600 µM LOR in the buffer solution (pH 7.0) at setting of the working electrode potential at 1200 mV *vs*. Ag/AgCl/KCl (3.0 M). (B) Cottrell’s plots for (a) 400, (b) 500, and (c) 600 µM LOR, based on the data obtained from the chronoamperograms

Chronoamperometric measurements of NOS and LOR at the MWCNTs/NADES/CPE were performed by setting the working e diffusion coefficient ode potential at 850 and 1200 mV *vs*. Ag/AgCl/KCl (3.0 M) respectively, for 0, 400, 500, and 600 µM NOS and the same concentrations of LOR in 0.05 M phosphate buffer (pH 7.0) (Figures 10A and 11A). Under the mass transport limited condition, for an electroactive substance (NOS and LOR in this case) with a diffusion efficient of D, the current related to the electrochemical reaction can be measured by the Cottrell equation (37):

I = nFAD^1/2^C_b_π^-1/2^t^-1/2^

 Equation 3.

where *D* is the diffusion coefficient (cm^2^ s^-1^), and *C*_b_ is the bulk concentration (mol cm^-3^). Experimental plots of *I* vs. *t*^-1/2^ were applied, with the best fits for NOS (Figure 10B) and LOR (Figure 11B). Using the obtained slopes and Cottrell equation, the mean quantities of the D were calculated to be 6.32 × 10^-6^ and 7.83 × 10^-7^ cm^2^ s^-1^ for NOS and LOR, respectively.

*Analytical aspects*

*Simultaneous determination of NOS and LOR*

**Figure 12:**
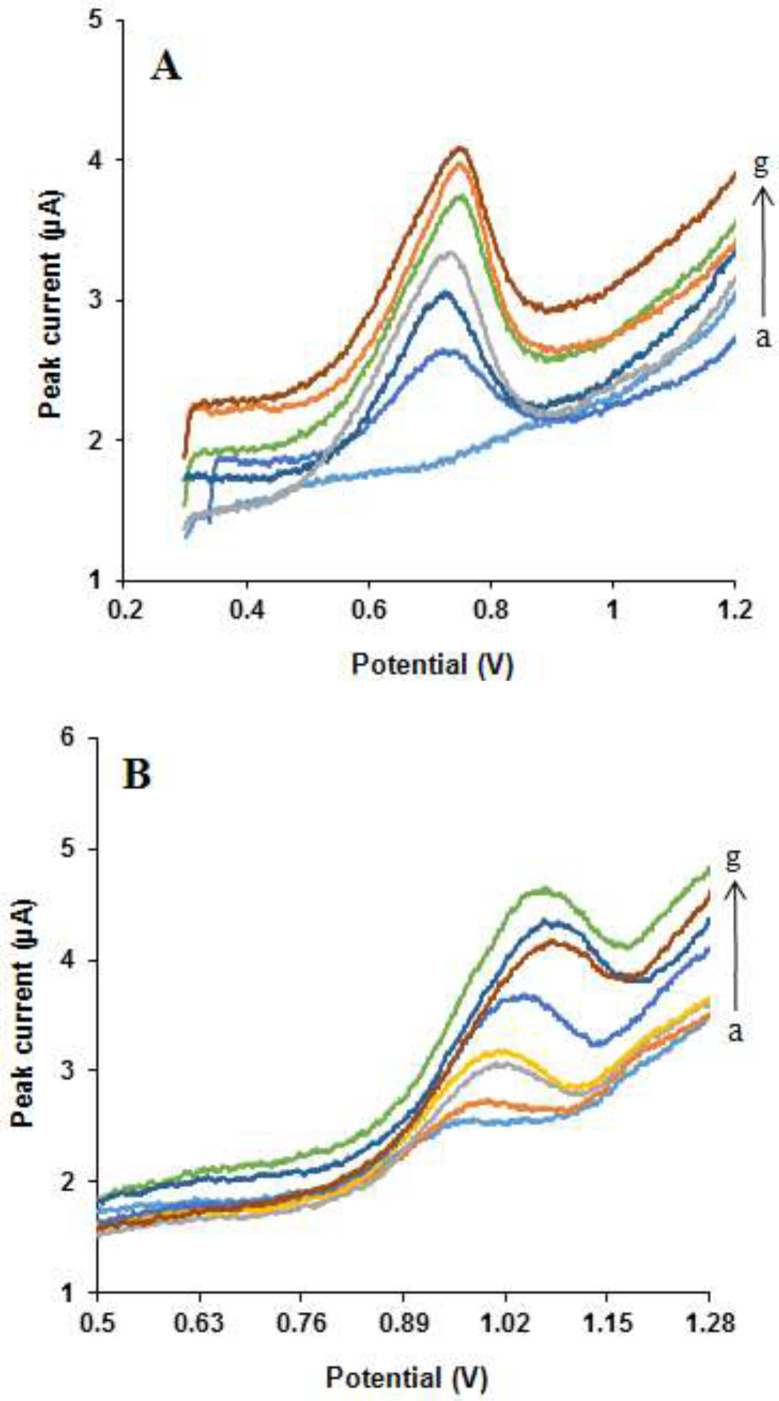
Typical cyclic voltammograms for determination of (A) NOS, at different concentrations: (a) 3, (b) 10, (c) 100, (d) 500, (e) 700, (f) 1000 and (g) 1700 μM in the presence of 50 μM LOR and (B) LOR in various concentrations: (a) 1, (b) 10, (c) 100, (d) 500, (e) 1000, (f) 1700 and (g) 2220 μM in the presence of 50 μM NOS, under the optimal conditions at scan rate 50 mV s^-1^

Due to the high detection sensitivity, the differential pulse voltammetry method was chosen to establish an analytical method for the detection of NOS and LOR. The peak currents of NOS and LOR oxidations at the MWCNTs/NADES/CPE surface at the buffer solution (pH 7.0) were employed to determine these compounds (Figure 12). Under the optimized conditions, the peak currents of NOS and LOR were found to be proportional to their concentrations over the range 3-1700 µM and 1-2220 µM, respectively. The equations for regression lines were *I*_pa_, µA = 0.0003 × [NOS], µM + 0.7848 (*R*^2^ = 0.999) and *I*_pa_, µA = 0.0004 × [LOR], µM + 0.6 (*R*^2^ = 0.9997). The detection limits (3σ) were 1.90 µM for NOS and 0.69 µM for LOR. In Table 1, some analytical parameters of this work are compared with some reported methods [41-49]. It can be observed from the results. This study has a suitable linear dynamic range and limit of detection. These amounts are comparable with amounts presented by other research groups for the detection of NOS and LOR. Also, this technique is simple and does not require costly equipment, whereas the other reported techniques such as HPLC and LLE-MS/MS require costly instruments, complicated tools, and hazardous solvents.

*R* *epeatability and stability of the electrode*

The repeatability of the MWCNTs/NADES/CPE was studied, and relative standard deviations (RSD) of 1.8% and 1.9% for ten consecutive determinations of 500 µM NOS and 500 µM LOR were obtained, respectively. When the MWCNTs/NADES/CPE was stored under dry conditions for 7 days, the oxidation peak currents of NOS and LOR in solution were reduced less than 5.2% and 4.9%, respectively. The results presented that the modified electrode has good repeatability and stability as a sensor for the determination of NOS and LOR. 

*Interference studies*

The effects of potential interferents on observed peak currents of NOS (500 μM) and LOR (500 μM) in the differential pulse voltammetry were evaluated at pH 7.0. The potential interfering substances were selected from the group of substances usually found with NOS and LOR in biological fluids and/or in pharmaceuticals. The highest concentration of the interfering substance, which caused an error analytical of less than ±10% for detecting NOS and LOR, was considered the tolerance limit. After the experiments, it was found that 100-fold of NaCl, 125-fold of KCl, 1300-fold FeCl_3_, 500-fold of B_2_, 1100-fold of B_1,_ and 300-fold of ascorbic acid did not interfere with the voltammetric signals of NOS. Also, it was confirmed that 250-fold of NaCl, 150-fold of KCl, 1000-fold FeCl_3_, 500-fold of B_2_, 1000-fold of B_1,_ and 1500-fold of ascorbic acid did not change the peak current of LOR. 

*Determination of NOS and LOR in real samples*

NOS and LOR were determined by the standard addition method in pharmaceutical samples on the MWCNTs/NADES/CPE under the optimized conditions. Results from NOS and LOR determination with recovery and precision are presented in Table 2. The relative standard deviation (RSD) was used for expressing the precision of the analysis. Also, as a recovery experiment, the accuracy was obtained by calculating the relative error between the measured mean and the concentrations declared in this method. The obtained results confirmed the analytical usefulness of the proposed method for the detection of NOS and LOR in pharmaceutical formulations.

**Table 1:** Comparison of some methods for determination of NOS and LOR with this work

**Method**	**Analyte**	**LOD (µM)**	**LDR** ^a^ ** (µM)**	**Ref.**
HPLC	NOS	–	84.66-507.96	(41)
HPLC-ESI-MS/MS^b^	NOS	1.0	12-120	(42)
ATR-FT-IR and FT-RS^c^	NOS	–	24.2-5560	(43)
LLE-MS/MS	NOS	0.24	0.24-242.1	(44)
Voltammetry^d^	NOS	0.4	1.0-35	(45)
RFIA^e^ spectrophotometry	LOR	1.90 and 7.31	6.23-124.55 and 77.84-1245.49	(46)
HPLC-DAD^f^	LOR	∼ 0.1	–	(47)
HPLC-UV^g^ and HPLC-ED^h^	LOR	0.11 and 0.29	–	(48)
ACDPS/HMDE^i^	LOR	0.059	0.16-3.58	(49)
DPV	NOS	1.90	3-1700	This work
DPV	LOR	0.69	1-2220	This work

^a^Linear dynamic range, ^b^liquid chromatography followed by electrospray mass spectrometry, ^c^Fourier transform infrared spectroscopy by a diamond composite ATR crystal and NIR-FT-Raman spectroscopy methods along with primary solid phase extraction, ^d^Using graphene nanosheets modified glassy carbon electrode,^ e^Reverse flow injection analysis, ^f^Diode array detector, ^g^High-performance liquid chromatography-ultraviolet detection, ^h^High-performance liquid chromatography-electrochemical detection, ^i^Adsorptive cathodic differential pulse stripping on a hanging mercury drop electrode.

**Table 2:** Determination results of NOS and LOR in pharmaceutical samples

**Sample**	**Labeled (µM)**	**Detected (µM)**	**Recovery (%)**	**RSD (%) (n = 3)**
NOS	50	46	92	0.37
NOS	100	93	93	0.081
NOS	150	140	93.3	0.060
LOR	50	53	106	0.024
LOR	100	95	95	0.040
LOR	150	140	94.7	0.060

## Conclusion

The MWCNTs/NADES/CPE was fabricated according to modifying CPE with multi-walled carbon nanotubes and NADES. The modified electrode was used as a sensitive sensor for simultaneous measurement of NOS and LOR. The MWCNTs/NADES/CPE significantly enhanced the oxidation peak currents for ACP and TSA at the modified electrode. The proposed method displayed suitable characteristics, such as simplicity, low cost, high sensitivity, rapid analysis procedures, and wide liner range. The modified electrode was successfully applied to determine the concentration of NOS and LOR in real samples. 

## Conflict of interest

The authors declare that they have no conflict of interest.

## Author contributions

All authors contributed equally to the manuscript. 
